# Multiphysics Modeling and Simulation of a Light-Controlled Variable Damping System

**DOI:** 10.3390/ma16083194

**Published:** 2023-04-18

**Authors:** Zhicheng Liu, Zhen Lv, Yujuan Tang, Xinjie Wang, Xiang Liu, Yusong Chen

**Affiliations:** 1 School of Mechanical Engineering, Nanjing University of Science and Technology, Nanjing 210094, China; zhichengliu@njust.edu.cn (Z.L.); zlv@njust.edu.cn (Z.L.); xiang.liu@airoha.com (X.L.); yschen@njust.edu.cn (Y.C.); 2 School of Intelligent Science and Control Engineering, Jinling Institute of Technology, Nanjing 211169, China; yjtang@jit.edu.cn

**Keywords:** light-controlled, damping, electrorheological fluid, PLZT ceramic

## Abstract

In this paper, a light-controlled variable damping system (LCVDS) is proposed based on PLZT ceramics and electrorheological fluid (ERF). The mathematical models for the photovoltage of PLZT ceramics and the hydrodynamic model for the ERF are established, and the relationship between the pressure difference at both ends of the microchannel and the light intensity is deduced. Then, simulations are conducted by applying different light intensities in the LCVDS to analyze the pressure difference at both ends of the microchannel using COMSOL Multiphysics. The simulation results show that the pressure difference at both ends of the microchannel increases with the increase in light intensity, which is consistent with results from the mathematical model established in this paper. The error rate of the pressure difference at both ends of the microchannel is within 13.8% between the theoretical and simulation results. This investigation lays the foundation for the application of light-controlled variable damping in future engineering.

## 1. Introduction

In recent years, variable damping technology based on smart materials, such as electrorheological materials and magnetorheological materials, piezoelectric materials, and shape memory alloys, has been widely used for vibration control, noise control, and fluid control [1]. However, the above-mentioned intelligent materials are all electromagnetic excitation intelligent materials, which need to use traditional electromagnetic excitation source devices and are prone to produce electromagnetic noise interference. Photorheological material is a kind of intelligent material with a rheological effect under the irradiation of light with a specific wavelength. Because the excitation source is light energy, it has the advantages of remote control and no electromagnetic interference [2]. Therefore, in some situations with high requirements for electromagnetic noise suppression, applying photorheological material in the field of variable damping control is expected to provide a method for light control variable damping. Unfortunately, the fluid viscosity in the photorheological material prepared at present changes slowly with light intensity [3]. Therefore, using a single photorheological intelligent material can not meet the needs of rapid light control of damping, but two or more smart materials can be combined to achieve light-controlled variable damping with the transformation of multiple physical fields.

Lanthanum-modified lead zirconate titanate (PLZT) ceramic is a special photoelectric intelligent material. It has the anomalous photovoltaic effect, pyroelectric effect, photothermal effect, thermal expansion effect, and piezoelectric effect under the excitation of an ultraviolet light source. From the perspective of photo-induced physical characteristics, high photovoltage (kV level) in the polarization direction of PLZT illuminated with UV light would be of great importance in the application of PLZT ceramic. In 2015, Huang et al. predicted a mathematical model for the photovoltage of PLZT using multi-physical field coupling [4]. In 2019, Jiang et al. used the photovoltage of PLZT ceramics to construct an electrostatic field to remove dust from lunar exploration equipment [5], confirming that PLZT ceramics can establish a photo-induced electrostatic field under light conditions. In 2020, Liu et al. applied the photovoltage of PLZT ceramics to an electrostatic structure and carried out a photoemission study on an electrostatic composite driver [6]. It is concluded that the technology using the photovoltage of PLZT to construct an electrostatic field is gradually maturing.

Electrorheological fluid (ERF) is a kind of smart rheological material, which is composed of dielectric particles of micro-nano size wrapped in insulating oil [7]. The rheological properties of ERF can be changed with external electrical stimulation [8], i.e., the viscosity of ERF increases significantly with the increase in electric field intensity if an external electric field is applied [9]. When the electric field intensity is high enough, the ERF can be transformed into an elastic solid, showing the properties of Bingham fluid. Extensive studies of damping tunable based on ERF have been carried out since ERF was first prepared by Winslow et al. in 1949 [10]. However, improving the ERF effect is still the key point for the application of ERF. In 2003, Wen et al. first discovered the giant ERF effect in the composite nano-particle system, and the shear strength of the ERF was greatly improved [11]. In 2016, Wu et al. successfully prepared a new nano-particle hybrid titanium calcium oxalate electrorheological material composed of micron-sized, spindle-shaped particles and nano-sized irregular particles. The giant ERF based on the composite material shows not only high yield stress but also low field-off viscosity, which greatly improves the electrorheological efficiency [12]. In 2018, Patil et al. applied the damping control technology of ERF to the semi-active intelligent rear seat artillery system [13]. Compared with the conventional rear seat system, the performance was significantly improved. Bai et al. applied ERF in the damping system of sandwich beams in the same year. They replaced the traditional damping control device with ERF, and the performance of the system was greatly improved [14]. However, the ERF must be exposed to an electric field to change the rheological properties. Traditional electromagnetic excitation devices will produce electromagnetic interference, which is not applicable in some situations with high requirements for electromagnetic interference.

Considering the deficiencies in ERF directly stimulated with external electric energy, a light-controlled variable damping system (LCVDS) is proposed. The electrostatic field generated by the photovoltage of PLZT ceramics is provided to the ERF, and the damping control can be realized without electromagnetic interference. In this study, the mathematical model of LCVDS is established, and the relationship between the pressure difference at both ends of the LCVDS and time under different illumination conditions is deduced. The fluid dynamics simulation of the ERF in the LCVDS is carried out using COMSOL Multiphysics.

## 2. Mathematical Modeling of a Light-Controlled Variable Damping System

### 2.1. Structure of the Light-Controlled Variable Damping System

**Figure 1:**
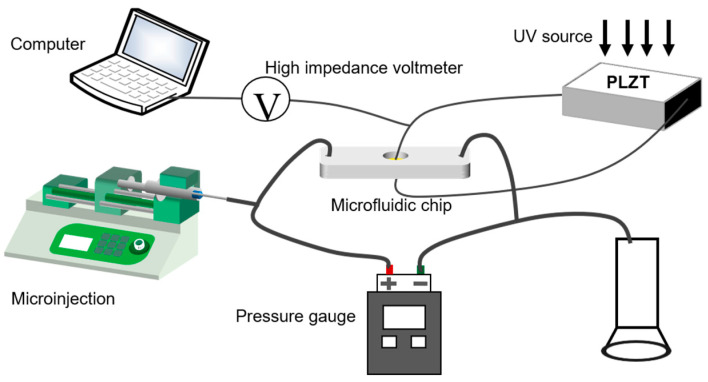
Light-controlled variable damping system.

The LCVDS is shown in Figure 1. When the PLZT ceramic is irradiated using a UV light source with a wavelength of 365 nm, a high photovoltage of PLZT ceramic will be rapidly generated. The PLZT ceramic and the microfluidic chip are connected with wires, and the photovoltage is transmitted to the upper and lower electrodes on the microfluidic chip to generate an electrostatic field. At the same time, the microinjection pump will transport the ERF to the microfluidic chip through the catheter at a constant speed. The pressure difference between the inlet and outlet of the microfluidic chip can be measured with the pressure gauge to analyze the influence of the electrostatic field generated by PLZT on the ERF.

**Figure 2:**
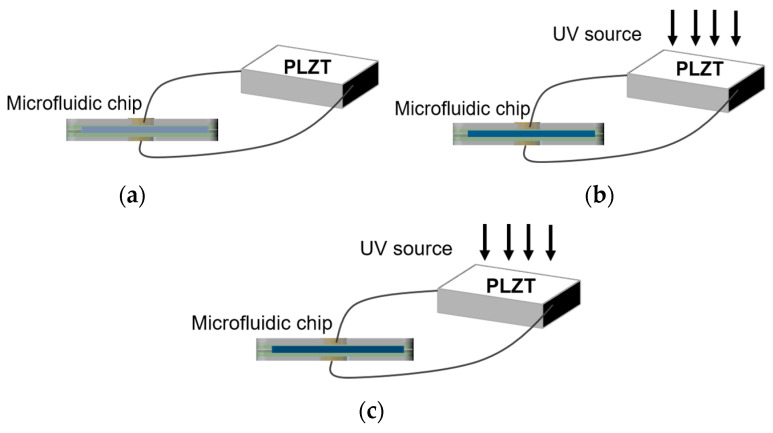
Schematic diagram showing ERF flow inside a microfluidic chip under different light intensities. (**a**) Light intensity of 0 mW/cm^2^; (**b**) light intensity of 50 mW/cm^2^; and (**c**) light intensity of 150 mW/cm^2^.

The microfluidic chip consists of seven layers, the upper and lower layers are the basement layers, the interior contains the electrode layer and the insulation layer, and the middle contains the channel layer that the ERF passes through. Since the photovoltage produced by the PLZT ceramic increases with the increase in light intensity, the damping force of the ERF can be regulated. As shown in Figure 2, with the increase in light intensity, the damping force in the ERF increases (color deepening).

### 2.2. Equivalent Circuit Model for the LCVDS

**Figure 3:**
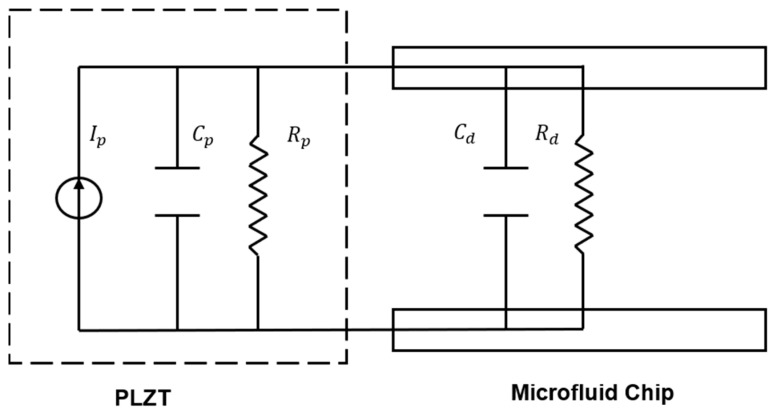
Equivalent circuit model for the LCVDS.

When the PLZT ceramic is illuminated using a UV light with a wavelength of nearly 365 nm, the photovoltage of PLZT ceramic is produced and applied to the upper and lower electrodes in the microfluidic chip with wires. Herein, the microfluidic chip of ERF is taken as the driven load of the PLZT ceramic in the LCVDS. Based on the equivalent circuit of PLZT ceramic illuminated using a UV light, the equivalent circuit model for the LCVDS consisting of a constant current source *I_p_*, the resistance of the PLZT ceramic *R_p_*, the capacitance of the PLZT ceramic *C_p_*, the resistance of the microfluidic chip *R_d_*, and capacitance of the microfluidic chip *C_d_*. This model is shown in Figure 3.

According to the equivalent circuit model in Figure 3, the expression for the photovoltage *Vp* of the PLZT ceramics is obtained as follows [15]:

(1) $$V_{P}=I_{P}\frac{R_{p}\cdot R_{d}}{R_{p}+R_{d}}\left(1-e^{-\frac{t}{\frac{R_{p}\cdot R_{d}}{R_{p}+R_{d}}\left(C_{p}+C_{d}\right)}}\right)=V_{s}\left(1-e^{-\frac{t}{\tau }}\right)$$

where, *V_s_* is saturated photovoltage, *V_s_* = $I_{P}\frac{R_{p}\cdot R_{d}}{R_{p}+R_{d}}$; *τ* is a time constant, and

$$\tau =I_{P}\frac{R_{p}\cdot R_{d}}{R_{p}+R_{d}}\left(C_{p}+C_{d}\right)$$

**Figure 4:**
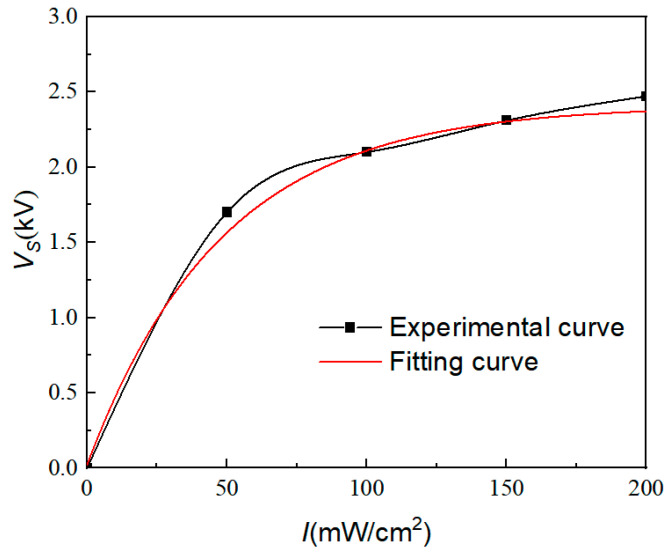
Relationship between light intensity and photovoltage.

In order to identify the parameters in Equation (1), the photovoltage of the PLZT ceramic connected to the microfluidic chip with ERF is measured first. In this section, the microchannel with the size of 6 mm (length) × 1 mm (radius) is filled with ERF. The PLZT ceramic (3/52/48) sample with the size of 15 mm × 15 mm × 1 mm was provided by the Shanghai Institute of Ceramics, Chinese Academy of Science. UV light with an intensity of 50 mW/cm^2^, 100 mW/cm^2^, 150 mW/cm^2^, and 200 mW/cm^2^ are applied on the PLZT ceramic, respectively, and the illumination time is set to 30 s. The photovoltage of PLZT ceramic with the microfluidic chip with ERF is measured using a high-impedance voltmeter (Trek 821HH), which is shown in Figure 4. The photovoltage of the PLZT ceramic is about 1.7 kV under the light intensity of 50 mW/cm^2^, which is a little larger than that reported in Reference [14] (about 1.5 kV under 50 mW/cm^2^). The photovoltage of the PLZT ceramic is about 2.5 kV under the light intensity of 200 mW/cm^2^, which is larger than that reported in Reference [5] (about 2.2 kV under 241 mW/cm^2^). It is noted that if the PLZT ceramics have different polarization conditions and dimension parameters, different photovoltage and photo-induced electric field intensity are produced when the PLZT ceramics is illuminated using ultraviolet light.

The expression between the photovoltage and the light intensity is obtained by fitting the curve:

(2) $$V\left(I\right)=-2.39e^{-\frac{I}{48.1}}+2.41$$

### 2.3. Fluid Dynamics Model for the ERF in the Microchannel

**Figure 5:**
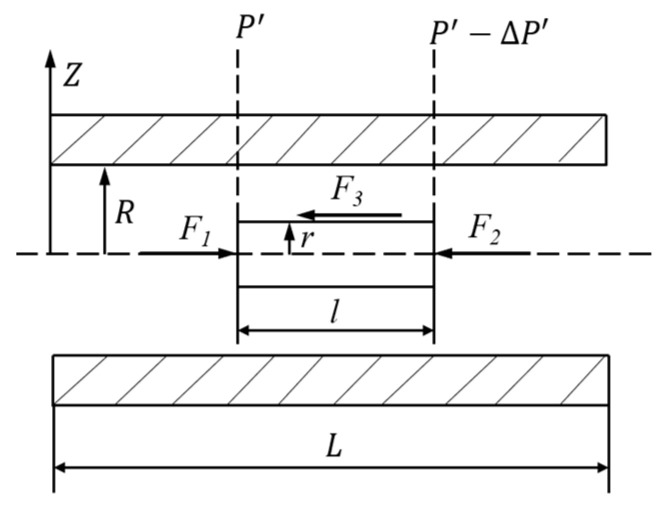
Stress in the ERF in the microchannel.

Without an electric field, the rheological properties of the ERF are similar to an ordinary fluid, which can be treated with the Newton fluid model. When the ERF is stimulated with an external electric field, the ERF shows an increase in viscosity and yield stress, which can usually be treated according to the Bingham fluid [16]. When the ERF flows in the microfluidic channel, the force analysis can be completed as shown in Figure 5. In Figure 5, the *L* is the length of the channel; *l* is the microelement length, *R* is the radius of the microchannel, and *r* is the microelement radius.

It can be seen from Figure 5 that the force balance can be written as:

(3) $$\sum F=F_{1}+F_{2}+F_{3}=0$$

**Figure 6:**
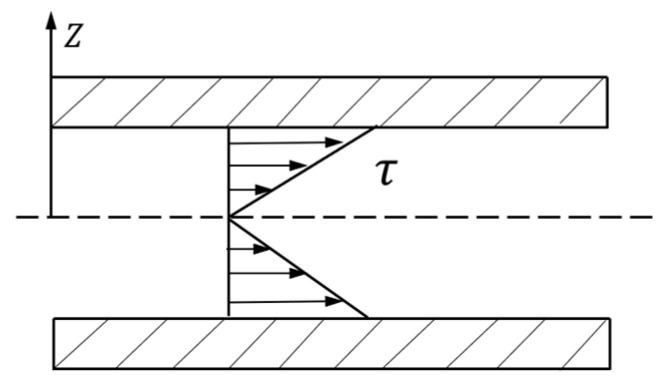
Shear stress distribution in the ERF.

According to the shear stress in Figure 6, Equation (3) can be rewritten as:

(4) $$\pi r^{2}P^{\prime }-\pi r^{2}\left(P^{\prime }-\Delta P^{\prime }\right)-2\pi r\tau \left(r\right)dl=0$$

Furthermore, Equation (3) can be simplified as follows:

(5) $$\tau \left(r\right)=\frac{r\Delta P^{\prime }}{2dl}=\frac{r\Delta P}{2L}$$

where Δ*P* is the pressure difference between the inlet and outlet of the microfluidic chip.

On basis of Equation (5), whether the liquid in the microchannel is a Newtonian fluid or Bingham fluid, the distribution of shear stress is proportional to the radius.

When there is no electric field, the ERF is similar to a Newton fluid and shows the properties of a Newton fluid. The constitutive model for the ERF without an external electric field can be expressed as follows [17]:

(6) $$\tau \left(r\right)=-\eta \frac{du\left(r\right)}{dr}$$

where, *η* is the viscosity of the ERF and *u*(*r*) is the velocity of the ERF.

Substituting Equation (6) into Equation (5):

(7) $$-\eta \frac{du\left(r\right)}{dr}=\frac{r\Delta P}{2L}$$

By integrating Equation (7), when *r* = *R* and *u* (*r*) = 0, we obtain the Poiseuille Equation of the radial velocity distribution of the ERF in the microchannel:

(8) $$u\left(r\right)=\frac{\Delta P}{4\eta L}\;\left(R^{2}-r^{2}\right)$$

**Figure 7:**
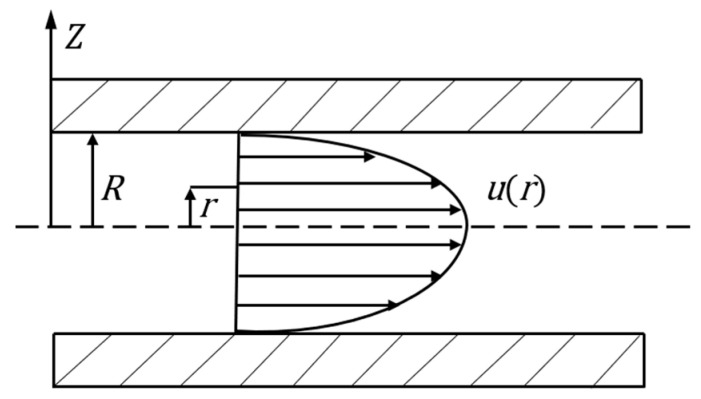
Velocity distribution of the ERF in the microchannel without an electric field.

The velocity distribution of the ERF in the microfluidic channel is shown in Figure 7.

According to Figure 7, the liquid flow in the microchannel can be obtained as:

(9) $$Q=2\pi \int_{0}^{R}u\left(r\right)rdr=\frac{\pi R^{4}}{8\eta L}\Delta P\;\;\;$$

Finally, the relationship between the pressure difference at the outlet and the inlet of the microchannel without an electric field can be obtained as:

(10) $$\Delta P=\left(P_{in}-P_{out}\right)=\frac{8\eta L}{\pi R^{4}}Q\;$$

where *P_in_* is the pressure at the entrance of the microchannel and *P_out_* is the pressure at the outlet of the microchannel.

**Figure 8:**
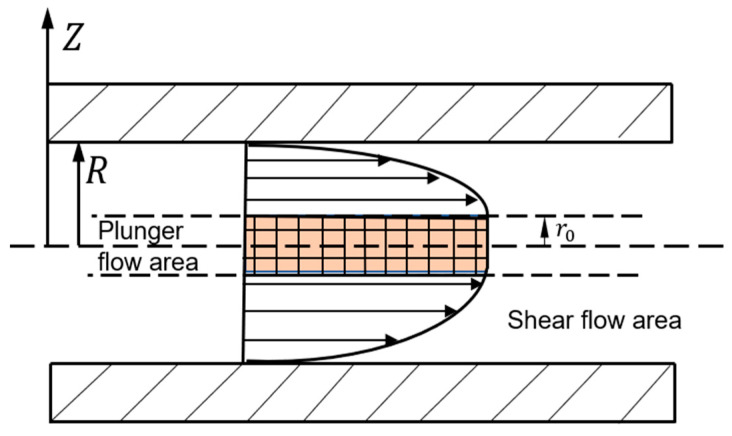
Velocity distribution of the ERF in the microchannel.

When the ERF is under an electric field condition, it shows the properties of a Bingham fluid. The plunger flow property is the most typical flow characteristic of the ERF. It can be seen from Figure 8 that the shear stress in the microchannel is the largest at the maximum radius, and the closer to the central area in the microchannel, the smaller the shear stress. When the shear stress generated by the pressure difference between the two ends of the microchannel at the maximum radius is smaller than the shear yield strength in the ERF, the ERF stays still. When the shear stress generated by the pressure difference between the two ends of the microchannel at the maximum radius is larger than the shear yield strength, the ERF near the maximum radius begins to flow, which is called the shear flow region, as shown in Figure 8.

In Figure 8, *r*_0_ is the radius of the cylindrical plug. The ERF can be divided into two parts, i.e., the plunger flow area (when *r < r*_0_) and the flow shear area (when *r > r*_0_).

In the area (*r*_0_ *< r < R*):

(11) $$\;\tau \left(r\right)=-\tau_{y}\left(E\right)+\eta \frac{du\left(r\right)}{dr}\;$$

When *u(r)|_r=R_ =* 0, we can obtain:

(12) $$\;u\left(r\right)=-\frac{\Delta P}{4\eta L}\left(R^{2}-r^{2}\right)-\frac{\tau_{y}}{\eta }\left(R-r\right)\;$$

In the area (0 < *r* < *r*_0_), as *du*(*r*)/*dr* = 0, *τ*(*r*_0_) = *τ_y_*, and by substituting Equation (6), we can obtain the radius of the cylindrical plug *r*_0_:

(13) $$\;r_{0}=\frac{2\tau_{y}L}{\Delta P}$$

It can be seen from Equation (11) that the radius *r*_0_ of the plunger area is inverse to the pressure difference Δ*P* at both ends of the microchannel. When the pressure difference Δ*P* is large enough, the plunger area will disappear. As *u*(*r*) = *u*(*r*_0_) = *C*, we can obtain:

(14) $$u\left(r\right)=-\frac{\Delta P}{4\eta L}\left(R^{2}-r_{0}^{2}\right)-\frac{\tau_{y}}{\eta }\left(R-r_{0}\right)\;$$

The velocity distribution of the ERF in the microfluidic channel is:

(15) $$\;u\left(r\right)=\left\{\begin{array}{c} \frac{\Delta P}{4\eta L}\left(R^{2}-r^{2}\right)-\frac{\tau_{y}}{\eta }\left(R-r\right)\;\;\;\;r_{0}<r<R\\ -\frac{1}{4\eta \frac{\Delta P}{L}}{\left[\left(\frac{\Delta P}{L}\right)R+2\tau_{y}\right]}^{2}\;\;\;\;\;\;\;0<r<r_{0} \end{array}\right.\;$$

The flow *Q* in the microflow channel is as follows:

(16) $$\begin{array}{c} Q=2\pi \int_{0}^{R}u\left(r\right)rdr=2\pi \int_{0}^{r_{0}}u\left(r\right)rdr+2\pi \int_{r_{0}}^{R}u\left(r\right)rdr\\ =\frac{\pi R^{4}\Delta P}{8\eta L}(1-\frac{8L\tau_{y}}{3R\Delta P}+\frac{1}{3}{\left(\frac{2L\tau_{y}}{R\Delta P}\right)}^{4}\; \end{array}$$

Integrating Equation (14), we obtain the following:

(17) $$\Delta P^{4}-\left(\frac{8\tau_{y}L}{3R}+\frac{8\eta L}{\pi R^{4}}Q\right)\Delta P^{3}+\frac{1}{3}{\left(\frac{2\tau_{y}L}{R}\right)}^{4}=0\;$$

When the flow *Q* is large enough, the above Equation can be simplified to:

(18) $$\Delta P=\left(P_{in}-P_{out}\right)=\frac{8\eta L}{\pi R^{4}}Q+\frac{8\tau_{y}L}{3R}\;$$

The relationship between shear yield stress *τ_y_* and the electric field can be expressed as [18]:

(19) $$\;\tau_{y}=kE^{\alpha }$$

where *α* is the field correlation coefficient and *k* is the amplification coefficient.

The final expression for the pressure difference Δ*P* at both ends of the microchannel is as follows:

(20) $$\;\;\Delta P=\frac{8\eta L}{\pi R^{4}}Q+\frac{8L}{3R}k{\left(\frac{V}{d}\right)}^{\alpha }$$

**Figure 9:**
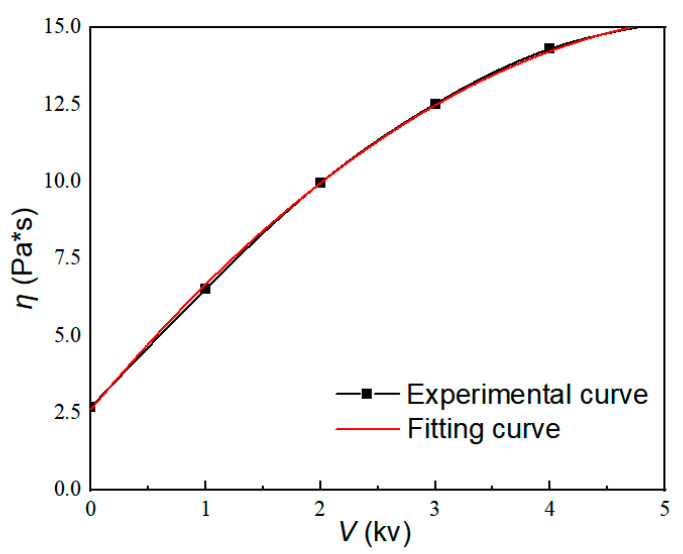
Relationship between viscosity and voltage.

According to Equation (20), the pressure difference Δ*P* is related to the flow *Q*, viscosity *η*, and yield stress *τ_y_* of the ERF. Furthermore, the *η* and yield stress *τ_y_* both vary with the electrical field that is applied to the ERF. In this section, the different voltages produced using PLZT ceramics with different intensities are applied to the upper and lower electrodes of the microfluidic chip to generate the electric field for the ERF. The viscosity properties of the ERF are measured using a Rheometer (Anton Paar MCR302) with the additional module for electrical field generation. The relationship between the viscosity of the ERF and the applied voltage is tested first, shown in Figure 9.

By fitting the experimental data in Figure 9, the expression between viscosity and voltage can be obtained as:

(21) $$\;\eta =-0.4V^{2}+4.4V+2.6$$

By substituting Equations (2) and (19) into Equation (20), the relationship between pressure difference Δ*P* and light intensity *I* is finally obtained as:

(22) $$\;\Delta P=\frac{8LQ}{\pi R^{4}}\left[-0.4V^{2}\left(I\right)+4.4V\left(I\right)+2.6\right]+\frac{8L}{3R}k{\left[\frac{V\left(I\right)}{d}\right]}^{\alpha }$$

**Figure 10:**
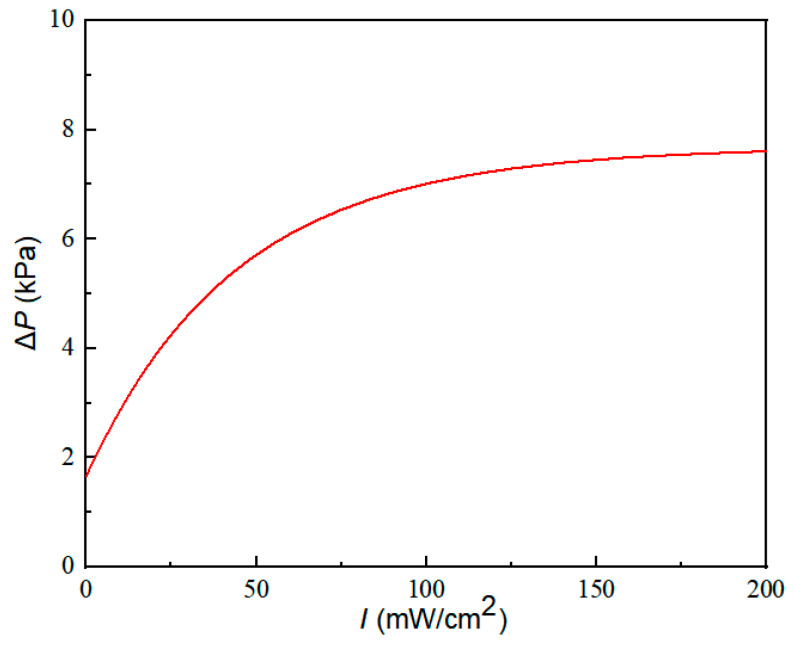
The relationship between the pressure difference at both ends of the microchannel and light intensity.

Referring to Equation (22), the pressure difference Δ*P* is finally related with the light intensity of the UV light. On basis of the parameters in Equation (22) and in Table 1, the relationship between the pressure difference Δ*P* and light intensity can be mathematically simulated, as shown in Figure 10.

It can be seen from Figure 10 that as the light intensity increases, the pressure difference Δ*P* at both ends of the microchannel also increases. When the light intensity is 0, the Δ*P* is not equal to 0, which is caused by the viscosity of the ERF itself. When the light intensity changes in the range of 0−100 mW/cm^2^, Δ*P* changes with the light intensity response being more intense; when the light is stronger than 100 mW/cm^2^, the response of Δ*P* decreases gradually.

## 3. Multiphysics Simulation of the Light-Controlled Variable Damping System

In order to further study the LCVDS based on PLZT and ERF, a series of multiphysics simulations under different light intensities were carried out using COMSOL Multiphysics 5.6.

**Figure 11:**
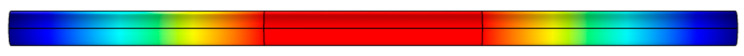
Electric field intensity distribution.

It was mentioned that in the LCVDS, the PLZT ceramic is electrically connected to the upper and lower electrodes of the microfluidic chip with wires to generate the electric field. In COMSOL Multiphysics, we load the photovoltage of PLZT ceramics (Equation (2)) in the middle region of the microfluidic channel, as shown in Figure 11.

**Figure 12:**
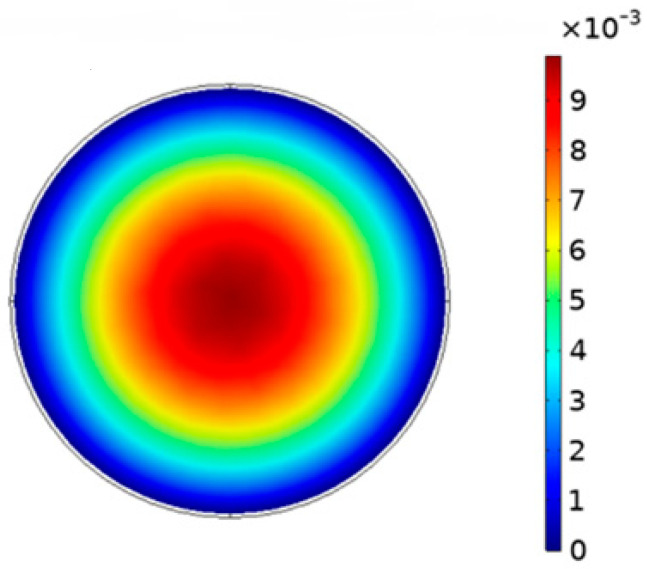
The flow velocity of the radial distribution.

The properties of the fluid have been set as the rheological properties of the ERF (Equation (21)) in COMSOL Multiphysics 5.6. The fluid is injected into the entrance on the left side of the microchannel at a flow rate of 5 mm/s. The velocity distribution of the electrorheological fluid in the microchannel is different because of the different distribution of the electric field in the microchannel, and the flow velocity of the radial distribution is shown in Figure 12.

**Figure 13:**
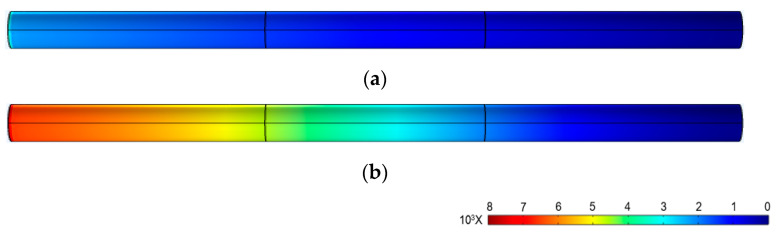
Pressure distribution in the microchannel. (**a**) The pressure distribution without photovoltage and (**b**) the pressure distribution with saturation photovoltage produced using PLZT ceramic under 200 mW/cm^2^.

In Figure 12, as the radius increases, the flow rate of the ERF slows down. When it is close to the inner wall of the microchannel, the flow rate is reduced to 0. In the middle area of the channel, the flow velocity of the ERF is the largest, and a plunger flow area is formed. The simulation result is consistent with the hydrodynamic model of the ERF. The simulation of the pressure difference in the microchannel is carried out to better analyze the pressure difference between both ends of the microchannel, as shown in Figure 13.

**Figure 14:**
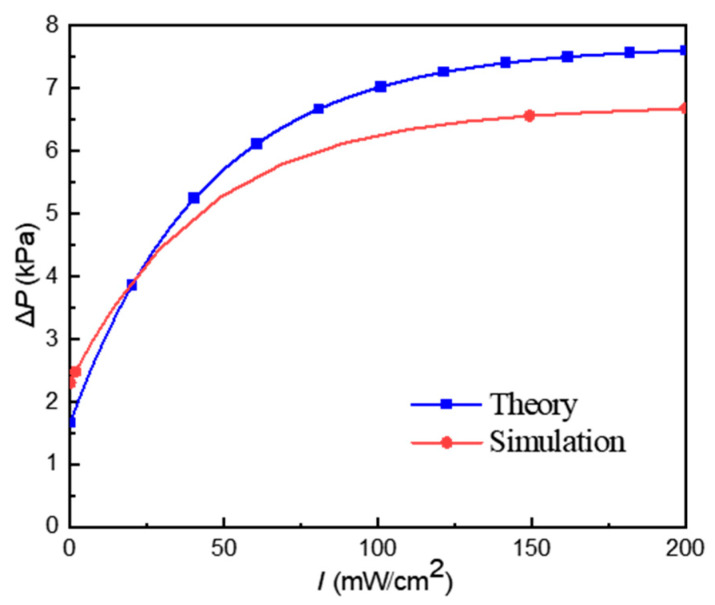
Comparison of the theoretical and simulation results.

In Figure 13, the pressure in Figure 13a,b at the outlet on the right side of the microchannel is 0 Pa since the outlet is connected to atmospheric pressure. The pressure at the entrance is the pressure difference on both sides of the microchannel. Comparing Figure 13a,b, we can find that the photovoltage produced using the PLZT ceramic has a great influence on the pressure difference at both ends of the microchannel. In order to further verify the rationality of the theoretical model, a comparison of the pressure difference between the theoretical (Figure 10) and the simulation results is conducted, as shown in Figure 14.

In Figure 14, there is an initial pressure difference at both ends of the microchannel when the light intensity is 0 mW/cm^2^, which is caused by the viscosity of the ERF itself. As the light intensity increases, the pressure difference at both ends of the microchannel gradually increases, and the rate of increase slows down gradually. This trend in the simulation curve is consistent with the trend in the theoretical curve. With the increase in light intensity, the pressure difference at both ends of the microchannel tends to be saturated. Furthermore, as the light intensity changes in the range of 0–200 mW/cm^2^, the error rate between the theory and simulation is within 13.8%, which further verifies the rationality of the mathematical model.

## 4. Conclusions

In this paper, a new method based on the photoelectric smart material PLZT ceramics and intelligent rheological material ERF is proposed to achieve light-controlled variable damping. When the PLZT ceramics are irradiated using UV light with a light intensity of 0−200 mW/cm^2^, a photovoltage of about 0–2.5 kV can be generated to construct an electrostatic field. The rheological properties of the ERF in the microchannel can be changed with the electrostatic field. We constructed a mathematical model for the photovoltage of PLZT ceramics and the hydrodynamic model of the ERF to obtain the relationship between the pressure difference at both ends of the microfluidic microchannel and the light intensity. The theoretical results show that the pressure difference at both ends of the microchannel increases with the increase in light intensity, where the pressure difference at both ends of the microchannel increases from about 1.7 kPa to 7.6 kPa as the light intensity increases from 0 to 200 mW/cm^2^. Simulations were conducted based on LCVDS to verify the correctness of the mathematical model. The simulation results show that the trend in pressure change is basically consistent with theory, and the pressure difference increases from about 2.3 kPa to 6.7 kPa as the light intensity increases from 0 to 200 mW/cm^2^. The correctness of the mathematical model was verified by comparing the theoretical and simulation results.

This method provides a more suitable damping regulation technology for situations with high electromagnetic interference requirements and addresses the disadvantage of slow response speed in traditional light-controlled variable damping. The advantages of excitation source cleaning and remote control can be achieved. In the future, the experimental research on LCVDS presented in this paper will be carried out to compare with the theory and simulations, and the preparation method of ERF will be further improved.

## Figures and Tables

**Table 1:** Physical parameters and geometric dimension values.

Parameter Name	Numerical Value
Channel length (*L*)	0.006 m
Flow (*Q*)	3.9 × 10^−9^ (m^3^/s)
Channel radius (*R*)	0.001 m
Amplification coefficient (*k*) [18]	100.68
Field correlation coefficient (*α*) [18]	1.57
Electrode plate distance (*d*)	0.003 m

## Data Availability

Data are available on request.
